# Identification of immune-infiltrated hub genes as potential biomarkers of Moyamoya disease by bioinformatics analysis

**DOI:** 10.1186/s13023-022-02238-4

**Published:** 2022-02-23

**Authors:** Fa Jin, Chuanzhi Duan

**Affiliations:** The National Key Clinical Specialty, The Engineering Technology Research Center of Education Ministry of China, Guangdong Provincial Key Laboratory On Brain Function Repair and Regeneration, Department of Cerebrovascular Surgery, Neurosurgery Center, Zhujiang Hospital, Southern Medical University, Guangzhou, 510282 China

**Keywords:** Moyamoya disease, Immune infiltration, Neutrophils, UNC13D, Bioinformatics analysis

## Abstract

**Background:**

Moyamoya disease (MMD) is a rare chronic progressive cerebrovascular disease. Recent studies have shown that autoimmune inflammation may also be an important pathology in MMD but the molecular mechanisms of inflammation in this disease are still large unknown. This study was designed to identify key biomarkers and the immune infiltration in vessel tissue of MMD using bioinformatics analysis.

**Methods:**

Raw gene expression profiles (GSE157628, GSE141024) were downloaded from the Gene Expression Omnibus (GEO) database, identified differentially expressed genes (DEGs) and performed functional enrichment analysis. The CIBERSORT deconvolution algorithm was used to analyze the proportion of immune cells between MMD and an MMD-negative control group. We screened for neutrophil-associated DEGs, constructed a protein–protein interaction network (PPI) using STRING, and clarified the gene cluster using the Cytoscape plugin MCODE analysis. The receiver operating characteristic (ROC) curve was applied to test and filter the best gene signature.

**Results:**

A total of 570 DEGs were detected, including 212 downregulated and 358 up-regulated genes. Reactome and KEGG enrichment revealed that DEGs were involved in the cell cycle, molecular transport, and metabolic pathways. The immune infiltration profile demonstrated that MMD cerebrovascular tissues contained a higher proportion of neutrophils, monocytes, and natural killer cells in MMD than in controls. The PPI network and MCODE cluster identified nine DEGs (*UNC13D*, *AZU1*, *PYCARD*, *ELANE*, *SDCBP*, *CCL11*, *CCL15*, *CCL20*, and *CXCL5*) associated with neutrophil infiltration. ROC results showed that *UNC13D* has good specificity and sensitivity (AUC = 0.7846).

**Conclusions:**

The characteristics of immune infiltration in the cerebrovascular tissues of MMD patients and abnormal expression of hub genes provide new insights for understanding MMD progression. *UNC13D* is shows promise as a candidate molecule to determine neutrophil infiltration characteristics in MMD.

**Supplementary Information:**

The online version contains supplementary material available at 10.1186/s13023-022-02238-4.

## Background

Moyamoya disease (MMD) involves the chronic progressive stenosis of the terminal part of the internal carotid artery and its main branches (middle cerebral artery, and anterior cerebral artery), which becomes an abnormal vascular network with smoke-like (Japanese: Moyamoya) compensatory capillary collaterals as an expression of pathologically increased angiogenic activity at the base of the skull [1, 2]. These vascular hallmarks are responsible for the main clinical features of the disease, which are recurrent ischemic and hemorrhagic strokes, often with serious consequences [3]. Epidemiologically, MMD has a higher prevalence in East Asia especially in Japan, South Korea and China (incidence: ≤ 0.94/100,000) than in Western countries [4, 5].

The etiology of MMD still remains unclear. Some reports indicate an association with autoimmune diseases, including autoimmune thyroid disease (Graves’ disease), type 1 diabetes, and systemic lupus erythematosus (SLE) [6–8]. Suzuki et al. [3] summarized the literature and found heavy deposition of IgG, IgM, and other immunoglobulins in the intimal thickening layer of MMD vessels. The most prominent pathological change in MMD is breach of the inner elastic lamina and the destruction and proliferation of smooth muscle cells in the tunica media [9], causing lumen narrowing or occlusion. Recent findings indicate that genetic factors may play a potential important role in the pathogenesis of the disease [10] and previous studies have reported up to 80% incidence of MMD in monozygotic twins [11]. In addition, MMD frequently occurs with inherited disorders, such as neurofibromatosis type I and Down syndrome [12, 13]. Using a genome-wide linkage and exome analysis, mutation in the ring finger protein 213 (*RNF213*) has been identified as the most critical susceptibility gene for MMD [14]. Additionally, other genome-wide association studies using single nucleotide polymorphisms (SNP) have revealed several susceptibility genes for MMD including *ACTA2*, *TGFB1, PDGFRB* and *RPTOR* [15, 16]. However, few studies have focused on the genetic alterations in vascular immune infiltration in MMD.

Bioinformatics analysis of gene expression profiles has played a critical role in studying the pathogenesis of human diseases in recent years. The use of gene chips may allow rapid detection of information about expression of all genes within the same sample time-point [17], and is a suitable approach for screening differentially expressed genes (DEGs). In this study, we used bioinformatics to analyze cerebrovascular tissue microarray data in MMD. The aim of the study was to identify MMD immune infiltration characteristics and specific DEGs, each of which may show promise as biomarkers or therapeutic targets, thus providing new insights into the pathogenesis of MMD.

## Methods

### Data collection and preprocessing

**Fig. 1:**
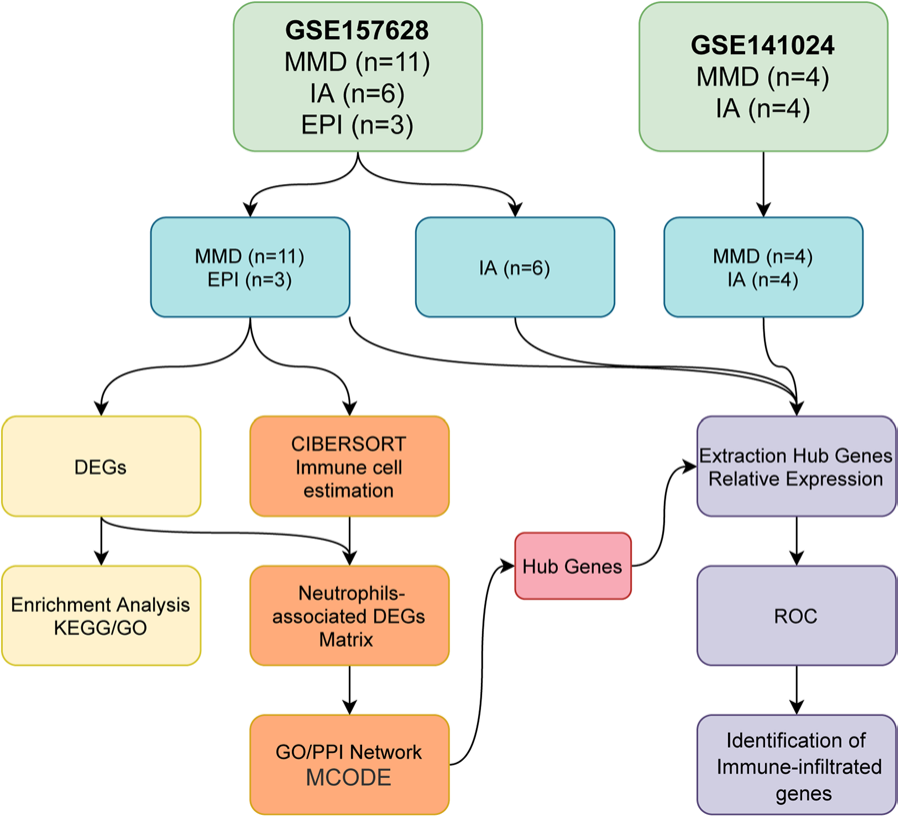
Workflow of the bioinformatics analysis

Figure 1 illustrates the workflow of this study. We searched for the keyword “Moyamoya disease” in the Gene Expression Omnibus (GEO) database to find and select datasets including data from vascular tissue in MMD and excluding small sample sizes, peripheral blood, and cerebrospinal fluid data. After removing duplicate subsets, the raw chip data of GSE157628 and GSE141024 were downloaded for analysis. There were generated from the same microarray chip with the chip model Agilent SurePrint G3 Human GE v2 8 × 60 K. GSE157628 contained middle cerebral artery (MCA) vascular wall tissue data from 11 patients with MMD, six patients with internal carotid aneurysm (IA), and three patients with epilepsy (EPI); the GSE141024 contained superficial temporal artery vascular tissue data from four patients with MMD and four patients with internal carotid aneurysm. Detailed sample information is shown in Additional file 1: Table S1. All samples were background corrected and quantile normalized using the Linear Models for Microarray Data (LIMMA) package [18] in Bioconductor before comprehensive analysis. We used the Data Table package to clean and extract the data. For probes with duplicate gene symbols, we used the mean as the unique expression value.

### Principal component analysis and DEGs screening

In order to select appropriate subgroups for DEGs analysis, we first visualized the distribution of the samples using principal component analysis (PCA) to assess the overall data patterns. MCA samples were selected from 11 MMD patients in the GSE157628 dataset as a case group and MCA samples from three EPI patients as an MMD-negative control group. The rationale for this approach was that MMD is a chronic vascular lesion with subtle variants from normal arteries in cellular composition, and that vascular tissue from EPI patients is more representative of normal vascular tissues than IA patients. The LIMMA package was used to identify DEGs using the criteria log fold change absolute value > 1 and *p* value < 0.01.

### Functional enrichment analyses of the DEGs

For functional enrichment analysis, Reactome and Kyoto Encyclopedia of Genes and Genomes (KEGG) [19] pathway enrichment analyses of the DEGs were performed using the Metascape [20] platform (http://metascape.org/gp). The gene symbol lists of up-regulated and down-regulated differential genes were uploaded to the server for analysis. All terms with enrichment significance of *p* < 0.05, count ≥ 5, and enrichment factor > 1.5 were selected automatically by the platform, based on their similarities. The results were imported into R and dot plots were created.

### CIBERSORT analysis

To quantify the proportion of immune cells in samples, we performed computational deconvolution using the CIBERSORT [21] web-based tool. A normalized gene expression matrix was used as input uploaded to the CIBERSORT server (https://cibersort.stanford.edu/). Both absolute and relative modes were applied, and quantile normalization was disabled. One thousand permutations were run for statistical testing. The result returned a percentage rate of immune cell types for all samples, and the sum of each sample’s immune cell ratio was 1.

### Extraction of mRNA expression data involved in the regulation of neutrophils

In order to clarify which DEGs are involved in regulating neutrophils, we first obtained gene symbol lists associated with the regulation of neutrophils from the AmiGO 2 website (http://amigo.geneontology.org/) by searching the keyword "neutrophils". Duplicate gene symbols were removed, and neutrophil-associated gene symbols intersected with DEGs were identified. Finally, we extracted an expression matrix based on intersection gene symbols from the normalized matrix.

### Gene ontology enrichment analysis

Gene Ontology (GO) is a major bioinformatics approach for the annotation of genes and analysis of their biological processes. We used Cytoscape software for network visualization (http://cytoscape.org/). The ClueGO plugin was used to analyze the neutrophil-associated genes through the gene ontology biological process (BP) analysis, and to establish a network relationship diagram.

### Protein–protein interaction network construction and hub gene identification

To gain insight into the genetic interactions related to the identified neutrophil infiltration, we used the STRING (https://string-db.org/) web-based tool [22] to analyze and construct a protein–protein interaction (PPI) network which would reveal the molecular mechanism by which neutrophil-associated genes are involved in MMD. The neutrophil-associated genes in the PPI network act as nodes, the line between two nodes represents the associated interaction, and the number of connections increases with the number of core genes. We visualized the PPI network result using Cytoscape software and used the Molecular Complex Detection (MCODE) [23] plugin to screen highly interconnected gene clusters in the PPI network. The criteria for selection in MCODE were as follows: MCODE scores ≥ 4, degree cutoff = 2, node score cutoff = 0.2, k-core = 2, and max depth = 100.

### Correlation analysis of hub genes and infiltrating immune cells

After identifying hub genes, we analyzed the relationship between the expression levels of hub genes and the proportions of infiltrating immune cells using Spearman’s correlation analysis in the ‘corrplot’ R package.

### Relative expression analysis of neutrophil-associated genes

To reduce bias caused by normalization of gene expression values, and to minimize false positives due to the use of p-value as a criterion for DEGs, we extracted the expression values of these hub neutrophil-associated genes and the internal reference gene (GAPDH) directly from the raw data. The relative expression of the genes was calculated to elucidate whether they were statistically different. All MCA samples from GSE157628 were used, including 11 cases of MMD, 6 cases of IA, and 3 cases of EPI.

### ROC analysis

To further test hub neutrophil-associated genes as potential indicators of MMD, we divided the 28 samples from GSE157628 and GSE141024 into two groups (MMD, non-MMD) according to their diagnostic information. We applied the receiver operating characteristic (ROC) curve and used the area under the curve (AUC) to evaluate diagnostic accuracy. The R package ‘pROC’ [24] was used to analyze results and visualize the data. The perfect AUC value is 1 and values over 0.5 are considered to have predictive value, with those closer indicating higher specificity and sensitivity.

### Statistical analysis

All statistical analyses were performed using R version 4.0.2. *P* values < 0.05 were considered statistically significant. Box, volcano, principal component analysis, bubble, and violin plots were drawn using the R package ‘ggplot2’ [25]. Heat maps were generated using the R package ‘pheatmap’ [26].

## Results

### Data preprocessing and DEG analysis

**Fig. 2:**
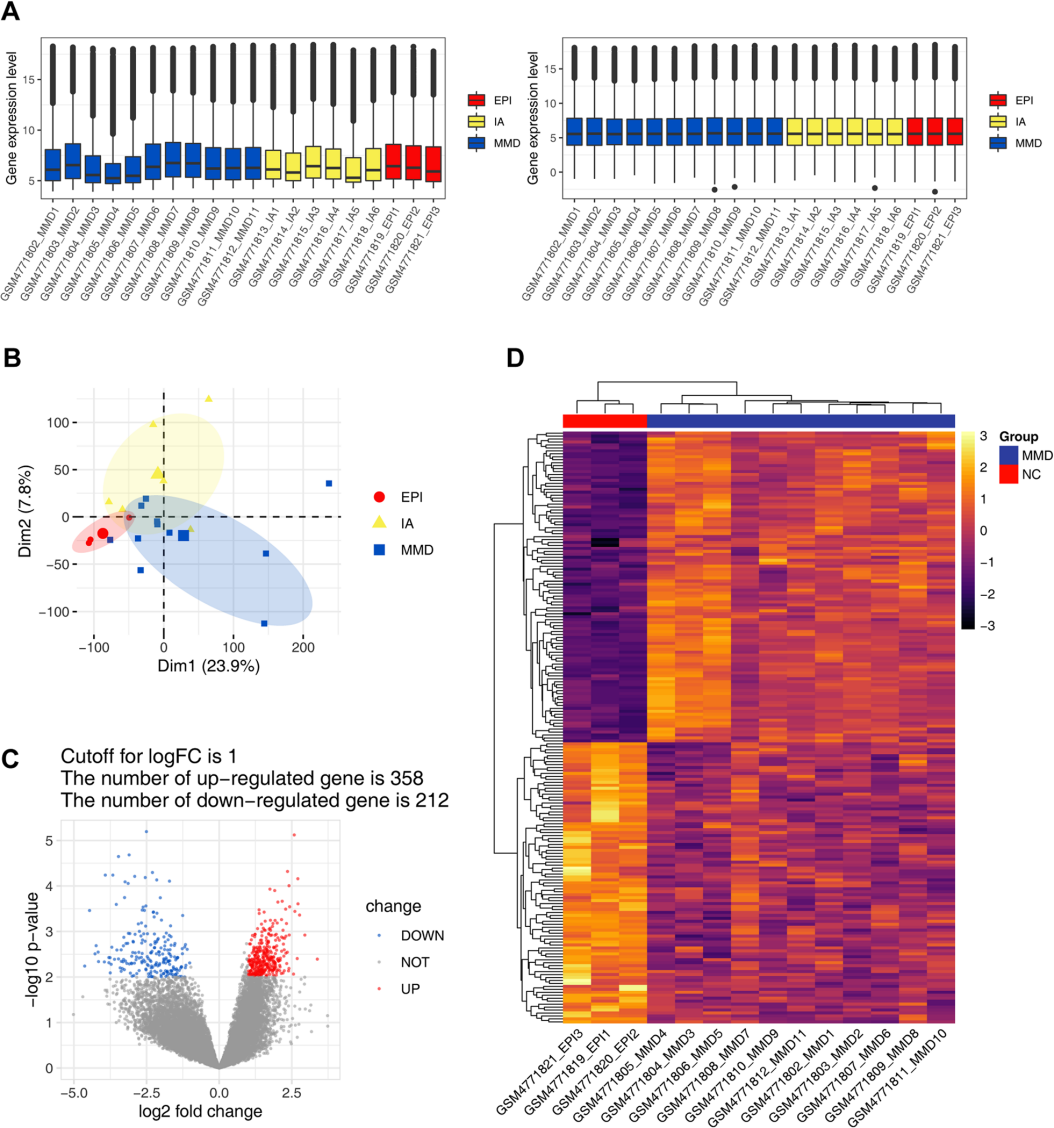
Normalization of all samples and DEG analysis. **A** Boxplots of sample expression, before normalization (left) and after normalization (right). The x-axis shows the sample information, and the y-axis the gene expression value. All samples are from middle cerebral artery vessel walls; blue (MMD) represents Moyamoya disease samples, yellow (IA) represents internal carotid aneurysm samples, and red (EPI) represents epilepsy samples. **B** PCA visualizes the grouping information of samples. **C** A volcano plot of data from all genes; each dot represents one gene. Genes that meet the screening criteria are shown in blue (down-regulated) and red (up-regulated), and the remaining genes are designated in gray. **D** A heatmap of the top 200 DEGs includes 105 up-regulated genes and 95 down-regulated genes. The first column shows the grouping information. Each row shows one gene, and each column shows data from one sample, including 11 MMD case samples and three EPI as negative control samples. Up-regulated genes are represented in bright colors, and down-regulated genes are represented in darker colors

After normalization of the expression matrix (Fig. 2A), we determined the grouping of sample data using PCA, and found no overlap in data distribution between the MMD and EPI groups, with good discrimination between the two; However, the IA group showed partial overlap with both the EPI and MMD groups, while the IA and EPI groups were clearly segregated (Fig. 2B). We considered that aneurysmal vessel walls generally have thrombus formation or inflammation due to eddy currents or rupture of the inner wall of the aneurysm, which leads to bias in the data. In addition, existing studies have shown that vessels from aneurysm patients are differentially expressed from normal vessels at the transcriptional level [27]. In order to identify the differential genes more precisely, we used only the EPI group as the negative control in the DEG analysis and excluded the IA group. Five hundred and seventy DEGs were screened out between the MMD and control groups, among which 358 genes were up-regulated and 212 genes were down-regulated. The volcano plot in Fig. 2C depicts the results of gene expression analysis. The 200 genes (Additional file 2: Table S2) with the most significant differences are shown in a heat map (Fig. 2D).

### Functional and pathway enrichment

**Fig. 3:**
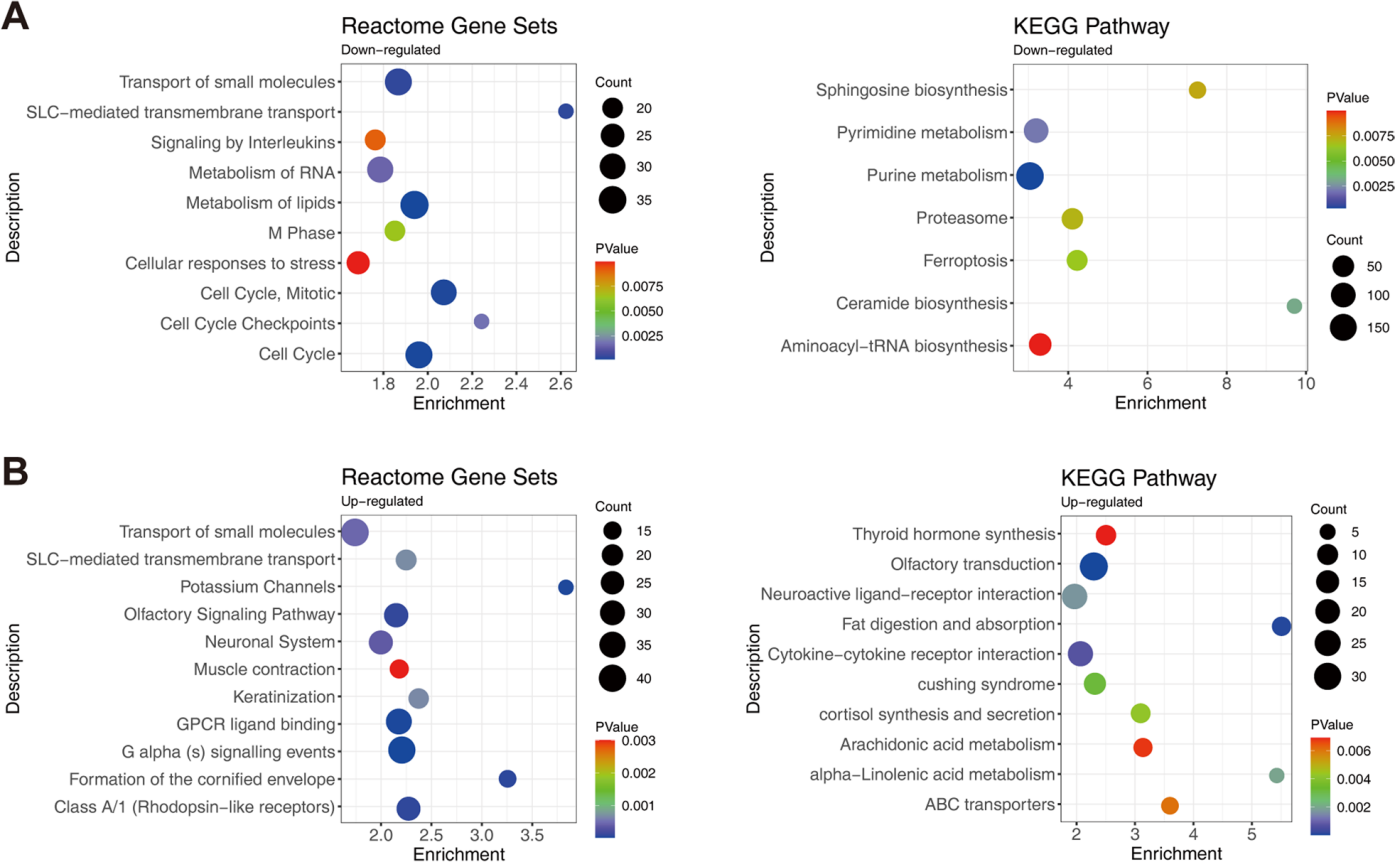
Functional enrichment analysis of DEGs. **A** Reactome pathway and KEGG pathway analysis of the down-regulated DEGs. **B** Pathway analysis of the up-regulated DEGs. Dot size represents the gene counts enriched in the term or signaling pathway. The color of each dot indicates the degree of significance

According to the Reactome gene sets enrichment analysis, there were 10 down-regulated terms and 11 up-regulated terms, mainly involving the small molecule metabolism and cell cycle biological processes. The results are shown in Fig. 3. Interestingly, in the Reactome enrichment results, both transport of small molecules and SLC-mediated transmembrane transport terms appeared in the up and down-regulated genes. The enrichment results of down-regulated DEGs in KEGG pathways were pyrimidine metabolism, purine metabolism, ferroptosis and others as shown in Fig. 3A. The enriched KEGG pathways of up-regulated genes were thyroid hormone synthesis, neuroactive ligand-receptor interaction, cytokine-cytokine receptor interaction, alpha-linolenic acid metabolism and others as shown in Fig. 3B.

### Immune infiltration analyses

**Fig. 4:**
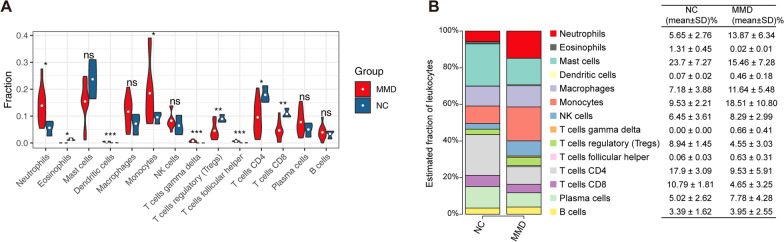
The landscape of immune infiltration between MMD and controls. **A** Violin chart of the ratio of immune cells. Y-axis is the fraction of immune cells; symbols indicate statistical significance between the two groups. ns: not significant; *p < 0.05; **p < 0.01; ***p < 0.001. **B** The bar graph on the left shows the percentage of immune sycell examples (mean) in samples from the negative control and MMD groups. Different colors represent immune cell populations; the table on the right shows the specific percentage values. NC: negative control, SD: standard deviation

Using the CIBERSORT algorithm, we obtained the immune infiltration of 14 immune cell subgroups in the MMD group and control group. Compared with the controls, the MMD group contained a higher proportion of neutrophils, monocytes, dendritic cells, gamma delta T cells, and follicular helper T cells, whereas the proportions of CD4 + T cells, CD8 + T cells, and eosinophils were lower than in controls (Fig. 4A, B).

### GO network analysis

**Fig. 5:**
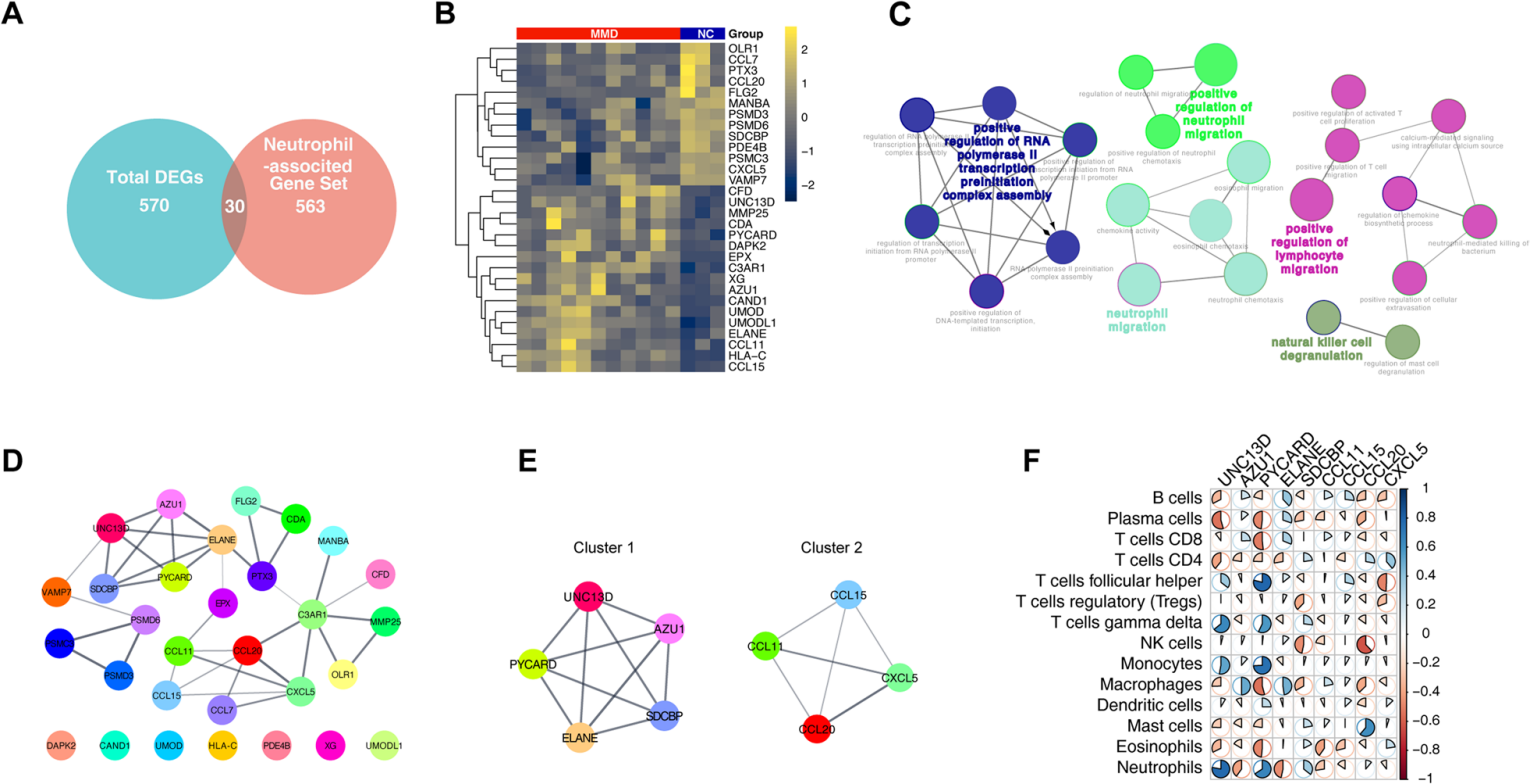
Identification of hub genes in neutrophil-associated DEGs. **A** Venn diagram representing the intersection genes taken between the differential gene set of MMD and neutrophil-associated gene set. A total of 30 intersection genes were identified. **B** Neutrophil-associated DEGs were extracted, and drawn as a heatmap. The heat map row names are the gene symbols intersected by the list of DEGs and neutrophil-associated genes. **C** Neutrophil-associated DEGs were subjected to GO functional enrichment analysis. The diagram shows the network between enriched GO terms, each dot being a pathway of enriched biological processes. The dot color indicates the terms of the cluster, and the most significant terms in the cluster are identified in bold font with color. **D** PPI network relationship graph of neutrophil-associated DEGs predicted by STRING. Each dot represents the protein molecule ultimately transcribed by each DEG and is distinguished by a different color. Connecting lines between proteins indicate the presence of their interactions, and the line thickness indicates the strength of interactions. The bottom row of dots shows protein molecules for which no network relationships were identified. **E** Identification of hub genes from the PPI network using MCODE. Cluster sub-networks were identified using Cytoscape’s MCODE plugin, and the top two cluster subnetworks are shown. Nodes and edges indicate the cluster members and their interconnections. **F** Heat map of the correlation between hub genes and immune cell ratios. The X-axis indicates the expression of hub genes; Y-axis shows the immune cell. Pie chart size represents the correlation coefficient, blue being positive and red negative correlation, color saturation indicating the strength of the correlation as shown on the bar to the right

Figure 5A shows the number of DEGs associated with neutrophil infiltration. These genes were all screened from the results of the difference analysis in Fig. 2. The expression characteristics of these neutrophil-associated DEGs are visualized on a heat map in Fig. 5B, and the network relationship between these biological processes is shown in Fig. 5C. GO analysis showed that these neutrophil-associated DEGs mainly involved RNA polymerase II transcription preinitiation complex assembly, regulation of neutrophil migration, regulation of lymphocyte migration, and natural killer cell degranulation. The detailed results are shown in Table 1.

**Table 1:** The enriched gene ontology terms for neutrophil-associated DEGs

Term	Description	Count	Genes	*P* value
GO:1990266	Neutrophil migration	10	C3AR1, CCL11, CCL15, CCL20, CCL7, CXCL5, DAPK2, PDE4B, UMOD, XG	7.33E−15
GO:0030593	Neutrophil chemotaxis	8	C3AR1, CCL11, CCL15, CCL20, CCL7, CXCL5, DAPK2, PDE4B	8.57E−12
GO:0072677	Eosinophil migration	5	CCL11, CCL15, CCL7, DAPK2, EPX	1.05E−09
GO:0008009	Chemokine activity	5	CCL11, CCL15, CCL20, CCL7, CXCL5	2.25E−08
GO:0048245	Eosinophil chemotaxis	4	CCL11, CCL15, CCL7, DAPK2	7.79E−08
GO:2000403	Positive regulation of lymphocyte migration	3	CCL20, CCL7, PYCARD	3.68E−05
GO:1902624	Positive regulation of neutrophil migration	3	C3AR1, DAPK2, XG	2.89E−05
GO:1902622	Regulation of neutrophil migration	3	C3AR1, DAPK2, XG	6.48E−05
GO:2000406	Positive regulation of T cell migration	2	CCL20, PYCARD	0.0012951
GO:2000144	Positive regulation of DNA-templated transcription, initiation	2	CAND1, PSMC3	0.0012951
GO:0090023	Positive regulation of neutrophil chemotaxis	2	C3AR1, DAPK2	0.0011382
GO:0070944	Neutrophil-mediated killing of bacterium	2	AZU1, ELANE	5.63E−05
GO:0060261	Positive regulation of transcription initiation from RNA polymerase II promoter	2	CAND1, PSMC3	7.89E−04
GO:0060260	Regulation of transcription initiation from RNA polymerase II promoter	2	CAND1, PSMC3	0.0013772
GO:0051123	RNA polymerase II preinitiation complex assembly	2	CAND1, PSMC3	0.0013772
GO:0045899	Positive regulation of RNA polymerase II transcription preinitiation complex assembly	2	CAND1, PSMC3	1.20E−04
GO:0045898	Regulation of RNA polymerase II transcription preinitiation complex assembly	2	CAND1, PSMC3	2.42E−04
GO:0045073	Regulation of chemokine biosynthetic process	2	AZU1, ELANE	3.61E−04
GO:0043320	Natural killer cell degranulation	2	UNC13D, VAMP7	7.50E−05
GO:0043304	Regulation of mast cell degranulation	2	UNC13D, VAMP7	0.0011382
GO:0042104	Positive regulation of activated T cell proliferation	2	EPX, PYCARD	7.89E−04
GO:0035584	Calcium-mediated signaling using intracellular calcium source	2	AZU1, CCL20	9.21E−04
GO:0002693	Positive regulation of cellular extravasation	2	ELANE, XG	7.27E−04

### Identification of hub genes using PPI network and modular screening

**Fig. 6:**
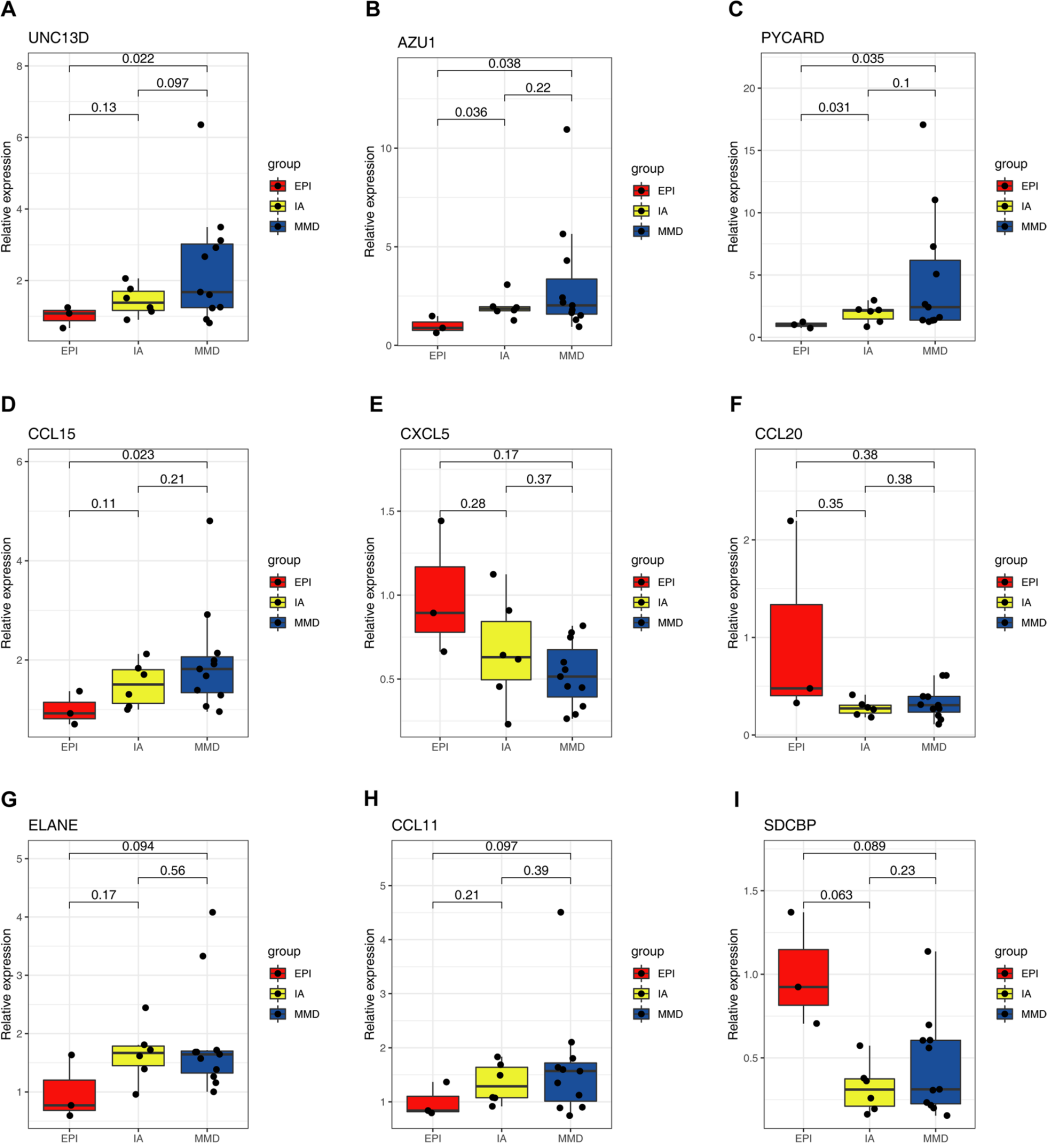
The relative expression of neutrophil-associated hub genes. **A**–**I** Expression values were extracted from the raw microarray data, and their ratio to the internal reference was calculated to compare the relative expression of hub genes in all samples of GSE157628. Three cases of EPI, six cases of IA, 11 cases of MMD, T-test, *p* < 0.05 was considered statistically significant

In order to clarify which are hub genes, we used the STRING database to perform PPI network analysis on 30 neutrophil-associated genes. The network diagram in Fig. 5D shows the results of network analysis and includes 37 edges and 23 nodes. Hub genes from the top two clusters included *UNC13D*, *AZU1*, *PYCARD*, *ELANE*, and *SDCBP* in cluster 1, and *CCL11*, *CCL15*, *CCL20*, and *CXCL5* in cluster 2 (Fig. 5E). Subsequently, correlation analysis showed that *UNC13D*, *PYCARD*, *SDCBP* and *CCL20* were positively correlated with neutrophils (*r* = 0.802, *r* = 0.690, *r* = 0.263, *r* = 0.107) while *AZU1*, *ELANE*, *CCL11*, and *CCL15* were negatively correlated with neutrophils (*r* = − 0.210, *r* = − 0.385, *r* = − 0.223, *r* = − 0.146) (Fig. 5F). The correlation results indicate that *UNC13D* and *PYCARD* have strong positive correlations with neutrophil infiltration. Finally, the relative expressions were calculated to examine the differences between these nine neutrophil-associated DEGs at the transcriptional level (Fig. 6A–I). The results showed that only *UNC13D*, *AZU1*, *PYCARD*, and *CCL15* were statistically significantly different between the EPI and MMD groups (*p* < 0.05). In addition, *AZU1* and *PYCARD* were statistically highly expressed in the EPI group vs IA group and EPI vs MMD group (*p* < 0.05).

### Diagnostic effectiveness evaluation

**Fig. 7:**
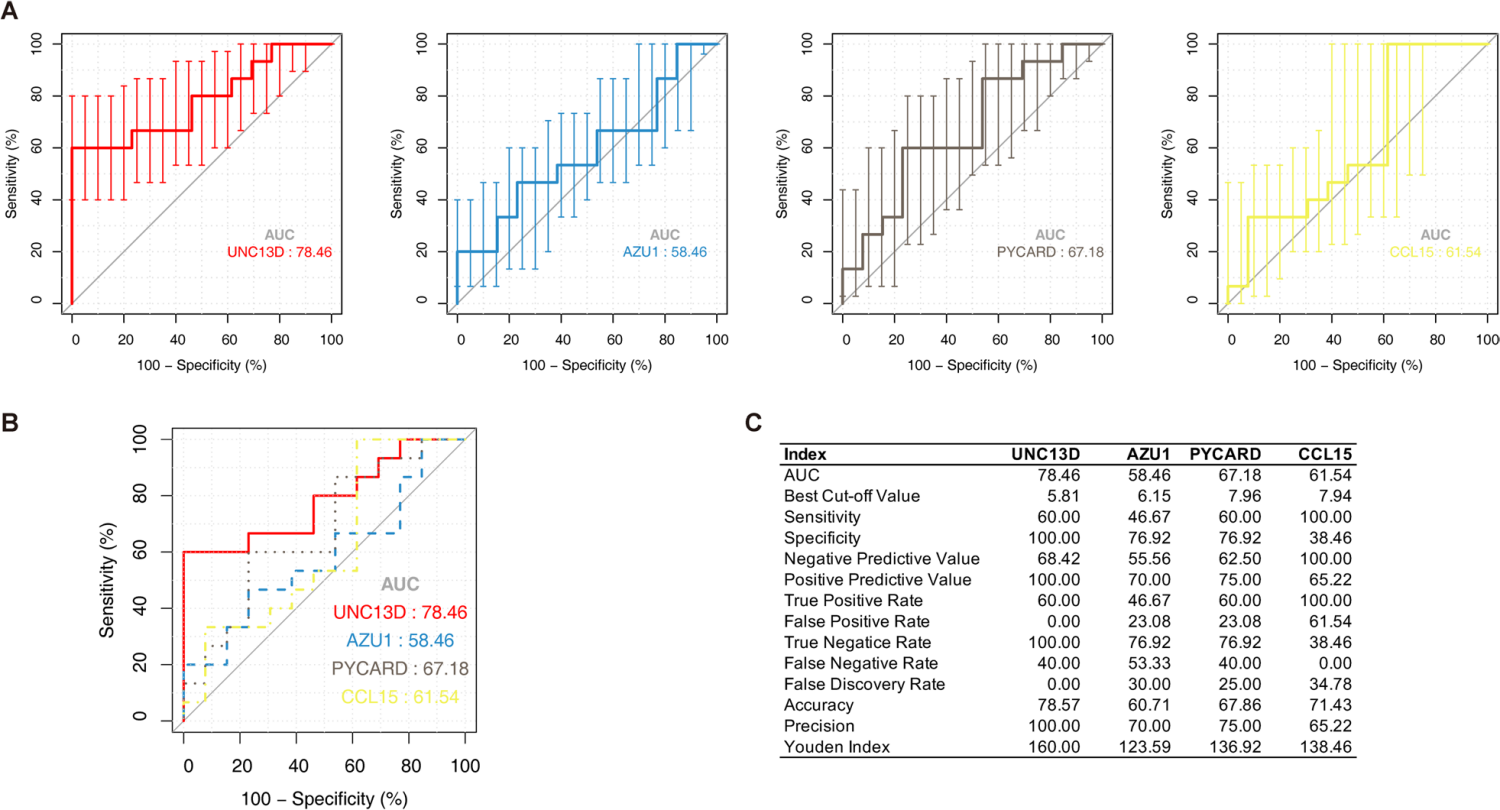
Test the candidate hub genes by receiver operating characteristic (ROC). **A** ROC curves for four candidate genes (*UNC13D*, *AZU1*, *PYCARD*, *CCL15*) are shown. Samples for ROC testing include all vascular tissue samples from the GSE157628 and GSE141024. The X-axis indicates specificity, the Y-axis indicates sensitivity, error bars are CI, and AUC represents the area under the curve. **B** Combined ROCs for comparison. **C** Table of detailed ROC analysis data

To assess the potential of four neutrophil-associated DEGs as biomarkers for MMD, we performed ROC analysis on these genes, and the four genes with results were used for visualization as ROC curves for comparison. ROC curve analysis confirmed that neutrophil-associated DEGs distinguish MMD from non-MMD with AUC between 0.5846 and 0.7846 (Fig. 7A, B), while the gene with the highest predictive power was *UNC13D* with an AUC of 0.7846. Detailed results are shown in Fig. 7C.

## Discussion

Our study is the first to use microarray data to reveal the characteristics of immune cell infiltration in the cerebrovascular system in MMD. Recent relevant genetic studies have used body fluid samples (cerebrospinal fluid or peripheral blood) from MMD patients to identify specific molecules such as autoimmune antibodies [28], transfer RNA-derived fragments [29], circular RNAs [30], long non-coding RNAs [31], and exosome-derived miRNAs [32]. We compared the immune infiltrating cell compositions in the samples to discover potential immune infiltration regulatory genes in MMD. Through further analysis, several hub genes involved in the regulation of neutrophil infiltration were identified. Thus, we conducted diagnostic ability tests on these newly discovered related genes to screen out the *UNC13D* as a novel biomarker.

Our enrichment analysis, suggested that small-molecule and transmembrane transport were enriched in down-regulated differential genes, as well as cell cycle changes. Interestingly, among the KEGG enriched pathways, we found up-regulated activation of pathways associated with thyroid hormone synthesis, in agreement with previous findings. Nakamura et al. [33] emphasized that excess thyroid hormone may alter cerebral hemodynamics, increase metabolism and oxygen consumption in the brain and be harmful to the arterial wall. Moreover, thyrotoxicosis may lead to hyperhomocysteinemia or sympathetic nervous activity, which is associated with premature atherosclerosis and MMD [33, 34].

MMD is closely related to chronic inflammatory cell infiltration. Masuda et al. reported that the primary component of intimal hyperplasia in the main internal carotid artery vessels of MMD patients is smooth muscle cells, with immune cell infiltration promoting their proliferation, leading to MMD vascular lesions [35], and indicating that immune-mediated pathological changes may be involved in the pathogenesis of MMD. However, due to methodological limitations, the landscape of immune infiltration in MMD remains unclear, particularly in subpopulations with a low abundance of cells. Our comparison of immune infiltration between MMD and non-MMD cerebrovascular tissues showed that increased neutrophil proportion was most significant in the up-regulated immune cells, which is noteworthy due to the important role of neutrophils in promoting vaso-occlusion and inducing angiogenesis [36, 37].

Neutrophils are innate immune phagocytes with a central role in immune defense, but they also damage host tissues through tightly regulated strategies of phagocytosis, degranulation, and release of neutrophil extracellular traps (NETs) [38]. The stimulation of neutrophils forms NETs via several proteins including NET activator serine protease neutrophil elastase (ELANE) [39]. NET release results in a cell death process known as NETosis [40], and participate in the pathological process of vascular occlusion: neutrophils accumulate in blood vessels in a P-selectin-dependent manner, they promote thromboxane A2vplatelet production, induce endothelial cells to express intercellular adhesion molecule 1 (ICAM1) [41], and strengthen the interaction between neutrophils and endothelium. The process triggers NETosis in vascular endothelial cells through a mechanism involving platelet-derived high mobility group box 1, reactive oxygen species, and integrin 1 [42, 43]. Conversely, the inflammatory signals initiating neutrophil recruitment trigger, neutrophils to secrete growth factors (such as VEGF-A, prokineticin2), chemokines, and MMP-9 to recruit other leukocytes to the damaged areas [44]. Research by Corey et al. [45] suggests that various neutrophil phenotypes facilitate an inherent autoimmune state in MMD patients, and crucially promote angiogenesis. On this basis together with the present microarray analysis results, we speculate that neutrophils may play a role in vascular occlusion and pathological changes to new capillaries in MMD.

The focus of this study was to explore the differences in gene expression and immune cell infiltration between MMD and a control group. We identified nine hub neutrophil-associated genes in total, of which *UNC13D* showed the highest diagnostic accuracy. *UNC13D* (protein unc-13 homolog D), also known as munc13-4 gene is one of the components of the neutrophil secretion mechanism [46], regulating neutrophil exocytosis (degranulation) in a calcium-dependent and SNARE protein dependent manner [47]. Under normal circumstances, neutrophil degranulation is tightly regulated because excessive particle content can cause tissue damage [48]. Consistent with this, in the present study, *UNC13D* was significantly up-regulated in MMD microarrays, a change which may be related to the increased degranulation of UNC13D regulated secretion, and whose mechanism needs to be verified experimentally. Additionally, a further eight genes were found to be involved in regulating neutrophils, as shown in Table 2.

**Table 2:** Characteristic of hub genes involved in the regulation of neutrophils

Gene symbol	Gene title	location	Function	*P* value
UNC13D	Unc-13 homolog D	Chromosome 17	Neutrophil degranulation	0.01615
AZU1	Azurocidin 1	Chromosome 19	Neutrophil degranulation	0.04886
SDCBP	Syndecan Binding Protein	Chromosome 8	Neutrophil degranulation	0.01608
ELANE	Elastase, neutrophil expressed	Chromosome 19	A serine protease secreted by neutrophils	0.04862
PYCARD	PYD And CARD Domain Containing	Chromosome 16	Neutrophil activation and degranulation	0.01679
CCL11	C-C motif chemokine ligand 11	Chromosome 17	Neutrophil recruitment	0.04675
CCL15	C-C motif chemokine ligand 15	Chromosome 17	Neutrophil recruitment	0.01857
CCL20	C-C motif chemokine ligand 20	Chromosome 2	Neutrophil recruitment	0.01271
CXCL5	C-X-C Motif Chemokine Ligand 5	Chromosome 4	Neutrophil recruitment	0.02993

There are several limitations to the present study. The number of samples we obtained from GSE157628 and GSE141024 was small, generating some bias when analyzing the DEGs and calculating the AUC, and more data samples are needed for validation in further research. Although this study confirms that *UNC13D* has good diagnostic properties and can be used as a potential biomarker for MMD, genetic diagnosis by obtaining vascular tissue is not feasible. Cerebral angiography, Computed tomography angiography or magnetic resonance angiography are more commonly used clinically for diagnosis. Due to the complex functions and molecular genetic mechanisms, these bioinformatics results need to be verified by experiments. The slow progression of MMD may have different gene expression at each time stage, the genetic characteristics of children and adults may differ, and these possible variations need to be further clarified.

## Conclusions

This study has provided new insights into the pathogenesis of MMD. We have revealed the composition of immune cell infiltration in the cerebrovascular tissues of patients with MMD for the first time using bioinformatics analysis. We have also identified nine hub genes related to neutrophil regulation, of which *UNC13D* may be a promising candidate biomarker for MMD.

## Supplementary Information

**Additional file 1: Table S1.** Samples information.

**Additional file 2: Table S2.** The top 200 DEGs as shown in Fig. 2 heatmap.

## Data Availability

The datasets analyzed in this study were downloaded and accessed from the Gene Expression Omnibus (GEO) database: https://www.ncbi.nlm.nih.gov/geo/, with accession No: GSE157628, GSE141024.

## Abbreviations

MMD: Moyamoya disease
GEO: Gene expression omnibus
DEGs: Differentially expressed genes
ROC: Receiver operating characteristic
AUC: Area under the ROC curve
miRNAs: Micro RNAs
SNP: Single nucleotide polymorphism
SLE: Systemic lupus erythematosus
MCA: Middle cerebral artery vascular
STA: Superficial temporal artery
IA: Internal carotid aneurysm
EPI: Epilepsy
PCA: Principal component analysis
KEGG: Kyoto Encyclopedia of genes and genomes
GO enrichment: Gene ontology term enrichment
PPI: Protein–protein interaction
NETs: Neutrophil extracellular traps
SMCs: Smooth muscle cells
